# Caring for Family Members With Alzheimer’s and Burnout Syndrome: Impairment of the Health of Housewives

**DOI:** 10.3389/fpsyg.2020.00576

**Published:** 2020-04-21

**Authors:** María Luisa Avargues-Navarro, Mercedes Borda-Mas, Alina de las Mercedes Campos-Puente, María Ángeles Pérez-San-Gregorio, Agustín Martín-Rodríguez, Milagrosa Sánchez-Martín

**Affiliations:** ^1^Department of Personality, Evaluation and Psychological Treatment, CTS-432 Research Team, University of Seville, Seville, Spain; ^2^Research Directorate, Inca Garcilaso de la Vega University, Lima, Peru; ^3^Department of Psychology, Universidad Loyola Andalucía, Seville, Spain

**Keywords:** burnout, family caregivers of Alzheimer’s patients, housewives, health, emotional disorders

## Abstract

Being a housewife may already be a psychosocial risk factor leading to chronic stress and burnout, and this may be aggravated when the housewife must also become the caregiver of a family member with Alzheimer’s. The burnout syndrome and how it can affect general health and the presence of emotional disorders were studied in housewives who were family caregivers of an Alzheimer’s patient. The sample selected was made up of 193 housewives, 96 of whom were also caregivers for a family member with Alzheimer’s. Sociodemographic measures used were the *Maslach Burnout Inventory* and *The General Health Questionnaire.* Burnout was found in a significant percentage of participants. Emotional exhaustion, effect on general health, and presence of emotional disorders were higher in caregivers. Emotional exhaustion, general health, and anxiety were more influential, while depersonalization affected the appearance of depressive symptoms more. Being a caregiver and emotional exhaustion appeared to be the best predictors of emotional disorders. It was confirmed that emotional exhaustion influenced appearance of anxiety and depression equally in both groups. In the case of caregivers, an exhaustion-illness spiral was produced. In this group, emotional exhaustion seemed to become more severe as a consequence of the presence of chronic illnesses, and possibly influence the number of hours spent on care and having children living at home. Future research should analyze in greater depth and in a larger sample, the role of these variables and widen the focus of attention to personal variables that could be acting as protective factors and could be subject to intervention. The discussion concludes with some actions that should be included in prevention programs for the groups studied.

## Introduction

At the present time, studies on the burnout syndrome have proliferated due to its growing prevalence and repercussions on both the person and the organization they work for. Burnout is understood as a three-dimensional syndrome characterized by emotional exhaustion, depersonalization, and limited personal accomplishment. It appears in response to chronic emotional stress, and is most frequent in persons who work in service occupations, caregivers, and in situations with strong emotional demand (Maslach and Jackson, 1981).

Scarce literature exist on housewives as a group. There has been a traditional lack of recognition of their labor or the associated psychosocial risks, among them stress and burnout (Pascual, 2001). However, the work they perform in the home, the number of family members they live with, and attention to minors or family members with health problems cause an effect similar to the working conditions under rotating shifts or night jobs, and overwork perceived in other professions. Therefore, the activity performed by these women could in itself be a psychosocial risk factor for the appearance of job stress and burnout syndrome, as well as consequences for their health. Thus, it has been demonstrated that the characteristics of the work done by housewives affect appearance of cardiovascular, immunological, gastrointestinal problems, back pain, low back pain, and others (Moral et al., 2011).

Apart from that, an aging population and increased life expectancy in today’s society have increased the presence of dependents in the home other than the children, such as Alzheimer’s patients, who have become more prevalent in recent years (Rogero-García, 2010). Specific studies on the prevalence of dementia in Spain have reported global rates ranging from 4.3 to 17.2%, and from 4 to 9% in most studies on patients over 65. Alzheimer’s disease is the most frequent type of dementia (from 50 to 70% of all cases) (Villarejo et al., 2019). At the present time, the life expectancy in Spain has already surpassed 85 for women and 80 for men (Instituto Nacional de Estadística (INE), 2017), which is among the highest, not only in Europe but also in the world. In addition, this country, like other Mediterranean European countries, such as Italy, Portugal, or Greece, is characterized by its family-based society, with strong relationships among its members, grounded in its family ideological values (Brenna and Di-Novi, 2016).

According to a recent report on the profile of aging in Spain (Abellán et al., 2019), which took into consideration life expectancy and sociocultural values, among others, the profile of the informal caregiver showed that women under 65 years of age, especially from 45 to 64, are responsible for over half the care load (in hours) compared to care provided by all caregivers (48.4%). In the case of elderly men who require assistance, it is mainly the wife, followed by a daughter, who are the caregivers. However, when it is elderly women who need help, the order is reversed, and it is the daughters who are most often the caregivers, followed by other family members or friends.

In most cases, it is the housewife, the woman who is devoted only to care of the home, who maintains a direct affective relationship with the patient, and the one who takes on the role of “informal” caregiver, often taking on the full care load (Gil et al., 2015), exposing her to additional stress marked by overwork and strong emotional demands, along with lack of recognition or help and the impossibility of changing the situation, which could explain the increased risk of burnout (Son et al., 2007; Moral et al., 2011; Pinquart et al., 2012; Rivera and Requena, 2013).

Studies done in this line with the main caregivers of Alzheimer’s patients have reported high indices of anxiety and more use of coping strategies directed at emotion, resulting in burnout (Papastavrou et al., 2007). The appearance of burnout also depends on how long they have been caring for the family patient and the severity of the dementia (Rivera and Requena, 2013; Iavarone et al., 2014).

In general, it has been found that emotional exhaustion of caregivers is significantly related to anxiety, and depersonalization with depressive symptomatology (Yilmaz et al., 2009; Chiu et al., 2015), although there are studies that also associate these depressive symptoms with emotional exhaustion (Truzzi et al., 2012).

The high stress of housewives in general, and in particular, those who must undertake the care of a dependent such as an Alzheimer’s patient, may affect various different areas of their lives, with important consequences to their health. Thus, the quality of their relationship with such relatives before their illness and the feeling of responsibility for taking over their care have been found to predict results in the health indicators of these women. Indeed, it has been shown that caring for persons with this type of dementia generates more wear and physical, psychological, and emotional deterioration in those bound affectively to the patient who must do so with very little training or preparation (Hodgins et al., 2011; Aldana and Guarino, 2012).

Similarly, health indicators are directly related to perceived overburden, such that the heavier the burden is perceived, the greater the deterioration in health. Specific studies done with family caregivers of Alzheimer’s patients, which evaluated the general health condition of the patients, found that around 65% of the cases evaluated needed specialized attention (Saavedra et al., 2013).

Findings suggest that the general health, and in particular, mental health of caregivers, is worse than the general population (Fernández de Larrinoa et al., 2011). Caregivers themselves specifically refer to problems related to anxiety and depression, such as exhaustion, backaches, headaches and muscle pain, sleep impairment, affectation of the immunological system, apathy and irritability, and so forth (Crespo et al., 2005; Lovell and Wetherell, 2011; Campos-Puente, 2016).

Although we focused on caregivers, studies suggest a heavier impact on health in general, and particularly, emotional disorders, among women with low self-efficacy, and those who furthermore, do not work outside of the home, that is, whose activity is limited to being a housewife (Pascual, 2001; Duggleby et al., 2016). Thus, informal caregivers show a higher risk of emotional disorders, such as anxiety and depressive symptomatology (Crespo et al., 2005).

Research has shown that both professional and informal caregivers have high scores on depressive symptomatology, mainly on state subscales. This shows that emotional affectation is temporary and associated with the specific conditions of the context and the caregiver role at that time (Cequera and Galvis, 2014). Although it cannot be affirmed that caregiving is the direct cause of the high scores on depressive symptomatology, its influence would probably be considerable because of its implications for potentially high stress (Fernández-Lansac and Crespo, 2011; Armon et al., 2014). In fact, depression levels in informal caregivers are associated with lack of resources and social support and perceived overburden due to the work of caregiving (Tuithof et al., 2015).

The women caregivers of family members with Alzheimer’s takes on their care out of love and moral obligation, even though it is an informal situation in their life cycle and upsets their plans for the future. Studies have shown that 37.7% of informal caregivers said they had no defined project in life beyond caregiving (Cequera and Galvis, 2014). Basically, older women who care for a husband with Alzheimer’s do so as their main function. However, the perception they have of the experience of caring varies significantly among caregivers. Studies such as one on caregivers of patients with dementia show a feeling of satisfaction associated with the quality of the relationship and the feeling of doing good work, that is, keeping the person cared for in good condition and giving them the best quality of life possible, as well as the self-esteem of the caregivers (Mackenzie and Greenwood, 2012; Lloyd et al., 2016). In other words, the feeling of acting according to the norms and moral values interiorized in their family model, thinking that they are doing what they should, could make them feel more satisfied with the work they are performing. However, that does not exempt them from the reality. That is, the satisfaction with that care is always going to be concomitant with stress and overburden, which is why there is a need to continue with research focused on these women.

In spite of the above, there are very few studies on housewives who perform their activity exclusively in the home, and among them, those who, in addition to this role, are the main caregivers of a family member with Alzheimer’s disease, without receiving any type of remuneration or support. This study was intended to prioritize a line of research that raises awareness of the reality these women live with, whose lives are focused only on caring for family and home, and how this situation is made even worse when they must also perform the role of caretaker of a family member with Alzheimer’s.

The first studies on this subject in Spain were done by Campos-Puente (2016) and Campos-Puente et al. (2019) demonstrated the high levels of emotional exhaustion in this group of women, and that emotional exhaustion predicts the amount and extent of diseases, somatic symptoms, and social dysfunction in them. Starting out from these preliminary findings and the needs observed in clinical practice, in which a larger number of women with anxiety and depression are attended to each year, we wanted to continue enlarging these preliminary findings by focusing on how each of the dimensions of the burnout syndrome is manifested in this group of women, as well as the impact that it could have not only on their general heath but also on the presence of emotional alterations, placing special emphasis on the role of emotional exhaustion (a major dimension of the syndrome) in the appearance of these alterations.

Our general objective was to analyze the presence of burnout and its relationship with the general state of health and presence of emotional disorders (anxiety and depression) in housewives (HW) and housewives who are also family caregivers of Alzheimer’s patients (HWC). It was also intended to find out whether the effect of emotional exhaustion on anxiety and depression differentiates between the group of HW and HWC, as well as what possible variables associated with their work as a housewife and caregiver could be significantly explained by emotional exhaustion in each group.

The final purpose was to determine the importance of this problem in the group subject of study so proposals for action can be integrated in social-healthcare intervention programs directed at developing general support policies for these women.

## Materials and Methods

A retrospective or post facto case/control design (Ato et al., 2013) was used to compare several general health variables in HW and HWC, particularly, the presence of emotional disorders (anxiety and depression).

### Participants

The sample was comprised of 193 housewives, distributed in two groups, HWC (*n* = 96) and HW (*n* = 97), who belonged to a federation of associations of Alzheimer’s patients and neighborhood associations in the south of Spain. The mean age was 49 (*SD* = 11.31; range = 20–80). Participant education levels were 59.6% primary school and 20.2% high school, and the rest had had some higher education (6.2% pre-university, 6.2% university, and 7.8% other courses), 78% were living with a partner (married or domestic partnership), and 22% had no partner (single, separated, divorced, or widowed), 87.6% had children, and of these, 51.3% were living with two or more of their children. The mean number of hours spent on housework was 15.20 (*SD* = 8.20); 46.6% of the participants had no chronic diseases and 37.8% did. Similarly, in the HWC group, 35.6% were the daughter of the patient cared for.

Criteria for inclusion were that they be women over 20 years of age whose only occupation was unremunerated work in the family home without any support. Further, caregivers had to be related to the patient, have been caring for their relative for over 2 years, and be the Alzheimer’s patient’s primary caregiver.

### Instruments

An *ad hoc* questionnaire consisting of 10 open and closed questions was prepared. Six referred to sociodemographic variables (age, sex, marital status, children, number of children, number of children living at home) and four to caregiving characteristics (relationship to patient, age, and number of hours caregiving per day). They were also asked whether they had any chronic diseases, and the number and type of disease were recorded.

The Spanish adaptation of the *Maslach Burnout Inventory (MBI)* (Maslach and Jackson, 1986) (Seisdedos, 1997) was used to evaluate burnout. The scale consists of 22 items and includes three dimensions: emotional exhaustion (EE), depersonalization (DP), and decreased personal accomplishment (PA). For classification into high, medium, and low burnout, the criteria used were those of the authors applied to the Spanish population sample (*n* = 1.138): EE (Low: <15; Medium:15-24; High: >24), DP (Low: <4; Medium:4-9; High: >9), and PA (Low: >39; Medium:33-39; High: <33).

The reliability indices in this sample, according to the Cronbach’s alpha coefficients, were 0.90, 0.79, and 0.71, respectively. Analyzed by groups, indices found were 0.81, 0.33, and 0.69 for EE, DP, and PA and in HWC, 0.88, 0.67, and 0.71, respectively. Thus, the instrument had highly acceptable convergent and discriminant construct validity for the total sample, but internal consistency in the depersonalization dimension could be improved in the group of housewives.

General health and presence of emotional disorders (anxiety and depression) were evaluated using the 28-item version of the Goldberg General Health Questionnaire (GHQ) (Goldberg and Hillier, 1979). The GHQ-28 contains four subscales: somatic symptoms, anxiety and insomnia, social dysfunction, and depression. The reliability indices for the subscales were satisfactory with a Cronbach’s alpha of 0.83, 0.85,0.74, and 0.82, respectively. Analyzed by groups, in HW, reliability indices found were 0.79, 0.79, 0.66, and 0.82 for somatic symptoms, anxiety and insomnia, social dysfunction, and depression and in HWC, 0.75, 0.83, 0.70, and 0.78, respectively. Thus, the instrument had highly acceptable convergent and discriminant construct validity both for the total sample and by group in all the dimensions of health.

### Procedure

Sampling was by convenience, by accessibility. When authorization was received from the centers, the researchers went to places in several different towns to meet with the women, either in groups or individually. At this time, they were explained the purpose of the study and that their participation was voluntary and anonymous, and they signed the informed consent document and filled out the evaluation instruments.

Researchers were given specific instructions on application of the test battery, the law on data protection, and ethical norms following the Helsinki Declaration.

### Statistical Analysis

#### Descriptive Statistics and Preliminary Analyses

Statistical analyses were done using the IBM SPSS Statistical Package for Windows.

The burnout variable was designed following the criteria described above (EE high >24; DP high >9; PA low <33. Descriptive analyses (frequencies, percentages, means, and standard deviations) were done for both HW and HWC in the various dimensions. All the participants answered all the questions adequately, so there were no missing data.

The relationship between being HW or HWC and the presence/absence of burnout was assessed by *Chi square* (χ*^2^)*. The Student’s *t* was used to test for the existence of any significant differences in the MBI and GHQ-28 dimensions between the HW and HWC groups. To find the effect size, the contingency coefficient *(r^2^_φ_)* was used with *Chi square*. Reference values were 0.1, 0.3, and 0.5 for small, medium, and large sizes, respectively (Blaikie, 2003). The Cohen’s *d* was calculated using the Lipsey and Willson (2001) formula, for the sample sizes in the two groups (HW/HWC) and Student’s *t.* Reference values for small, medium, and large sizes were 0.20, 0.50, and 0.80, respectively (Cohen, 1992).

When their general health, and specifically, the presence of anxiety and depressive symptomatology, had been analyzed, a correlation analysis was done using the Pearson’s *r* to analyze the relationship between general health and anxiety and depression levels and burnout. Reference values of 0.10–0.30, 0.30–0.50, and >0.50 were used for small, medium, and large sizes, respectively (Cohen, 1992). Then, to find out the weight of each dimension in the syndrome and activity performed exerted on general health and specifically on anxiety and depression, three multiple linear regression analyses were done using the “enter” method, with emotional exhaustion, depersonalization, personal accomplishment, and activity performed as the independent or predictor variables, and as dependent or criterion variables, the total general health score, and anxiety and depression subscale scores, respectively. The *f*^2^ was calculated to test the effect size in the regressions (Selya et al., 2012), using an online calculator (Soper, 2020). Reference values of ≥0.02, ≥0.15, and ≥0.35 were used as small, medium, and large sizes, respectively (Cohen, 1988).

### Primary Analyses

#### Multigroup Structural Equation Modeling

To test whether the effect of emotional exhaustion on anxiety and depression was similar in HW and HWC, a sequence of nested models ranging from an unconstrained multisample model with the parameters freely estimated across subsamples to more parsimoniously nested models that included different levels of equality constraints, was calculated using MPlus7 (Muthén and Muthén, 1998–2012). As a preliminary step, the fit of a general model to the whole sample was tested separately for each group (HW and HWC), with no invariance. This step showed us possible differences between the groups that had to be considered for the unrestricted model. Then, the following models were estimated (Lippke et al., 2007): (a) Model 1: non-invariance, unconstrained model (unrestricted model); (b) Model 2: equal factor loading across the subsamples (measurement equivalent model); (c) Model 3: Model 2 constraints plus equal factor variance and covariances; (d) Model 4: Model 3 constraints plus equal regression paths; and (e) Model 5: Model 4 constraints plus equal factor residuals (fully constrained). As the model posed was made up of three latent variables, one of them with quantitative items and two with categorical items, structural equation models were analyzed using WLSMV with parameterization Theta (Byrne, 2012).

Compliance with invariance was tested in each of the models, assuming a higher level of invariance every time, by analyzing overall model fit and increase in the Comparative Fit Index (CFI). The overall fit of the resulting models were assessed by checking whether the CFI and Tucker and Lewis Index (TLI) were from 0.90 to 0.95, whether the root mean square error of approximation (RMSEA) was from 0.06 to 0.08, whether the Test of Approximate Fit of RMSEA was non-significant, and whether the weighted root mean square residual (WRMR) was under 1.00 (Hu and Bentler, 1999; Hooper et al., 2008; Kline, 2010; DiStefano et al., 2017). To compare nested models, the fit of each more parsimonious model (i.e., invariance constraints imposed) was compared with the fit of the unrestricted model. As indicators of model invariance, we examined the change in CFI, which should be ≤0.01 (Cheung and Rensvold, 2002). When full invariance was not satisfied, partial invariance was explored. To determine the source of non-invariance, modification indices were rechecked and, if necessary, equality constraints imposed on the potential non-invariant parameters were freed to vary between groups.

#### Predictors of Emotional Exhaustion

To explore which quantitative variables could be affecting EE in both groups, a separate linear regression analysis was done for each group, with the hours spent on tasks and age as predictor variables, using the “enter” method. A factorial ANOVA was also done to check the effect on emotional exhaustion of the categorical variables, number of children living at home (none, one, two, three, or more) and number of chronic diseases (none, one, two, or three) in the HW group and the same plus the relationship to the person cared for (daughter, wife, daughter-in-law, granddaughter) in HWC. The effect size was analyzed using the Partial Eta Squared (*η^2^_*p*_*), with 0.01, 0.06, and 0.14 as small, medium, and large sizes, respectively (Cohen, 1988).

## Results

### Descriptive Statistics and Preliminary Analyses

The prevalence of burnout in the sample of women participating was 11.9%. When the two groups were compared, it was found that 5.7% of HW had burnout, a percentage only slightly surpassed in the HWC group (6.2%). However, the chi-squared test did not show any statistically significant between-group differences (χ*^2^* = 0.062; *p* = 0.800, *r^2^_φ_* = 0.018).

The scores on the syndrome dimensions, as observed in Table 1, show a mean EE of 27.78 for all the women participating, which is very high for this dimension. The mean DP was 7.21, which is medium, and the mean PA was 34.28, also corresponding to a medium level in this dimension.

**TABLE 1:** Mean scores and standard deviations in burnout syndrome dimensions.

	**Total (*N* = 193)**		**^(a)^HW (*n* = 97)**		**^(b)^HWC (*n* = 96)**		**Comparison**		
				
	***M***	***SD***	***M***	***SD***	***M***	***SD***	***T*_(191)_**	***p***	***Cohen’s d***
Emotional exhaustion EE (Low: <15 Medium: 15–24 High: >24)	27.78	14.92	21.71	11.90	33.9	15.21	6.205	<0.001	0.89*_*L*_*
Depersonalization DP (Low: <4 Medium: 4–9 High: >9)	7.21	6.43	7.10	5.41	7.31	7.35	0.225	0.820	0.03*_*N*_*
Personal accomplishment PA (High: >39 Medium: 33–39 Low: <33)	34.28	8.99	30.05	9.00	33.51	8.97	*t*_(190,99)_ −1.19	0.230	0.39*_*S–M*_*

*^(*a*)^Housewives; ^(*b*)^Housewives caregivers. *N*, null effect size; *S*, small effect size (*d* = 0.20); *M*, medium effect size (d = 0.50); L, large effect size (d = 0.80).*

The Student’s *t*-test for equality of means only showed statistically significant differences between the HW and HWC groups for EE (*T* = 6.205, *p* = 0.000, *d* = 0.89), where emotional exhaustion was higher in HWC than in HW. However, no significant differences were found in DP (*T* = 0.225, *p* = 0.820, *d* = 0.03) or PA (*T* = −1.19, *p* = 0.230, *d* = 0.39) (see Table 1).

Focusing on the general health of the participants and the presence of emotional disorders, the frequencies and percentages for the general health of the participants are shown in Table 2 by whether or not health of the total sample was affected and by activity performed. The results showed statistically significant between-group differences (χ*^2^* = 28.06, *p* = 0.000, *r^2^_φ_* = 0.359), indicating that the general health of the HWC group with 40.9% was more affected than in HW with 22.8%.

**TABLE 2:** Affectation of general health of participants.

	**Total (*N* = 193)**		**^(a)^HW (*n* = 97)**		**^(b)^HWC (*n* = 96)**		**Comparison**		
				
	**Fr**	**%**	**Fr**	**%**	**Fr**	**%**	***χ ^2^***	***p***	***r^2^_φ_***
Affected (≥5)	123	63.7	44	22.8	79	40.9	28.062	<.001	0.359*_*M*_*
Not affected (≤4)	70	36.3	53	27.5	17	8.8			

*^(*a*)^Housewives; ^(*b*)^Housewives caregivers. *N*, null effect size; *S*, small effect size (*r^2^_φ_* = 0.10); *M*, medium effect size (*r^2^_φ_* = 0.30); *L*, large effect size (*r^2^_φ_* = 0.50).*

In addition, to show the differences in general health and the anxiety and depression subscales by activity performed, first descriptive statistics are given for each of the subscales (including anxiety and depression) and the total score in general health based on the GHQ-28 questionnaire (see Table 3). Both on the subscales and on the total score in general health, the HWC group means were statistically significantly higher (*T* = −8.455, *p* = 0.000, *d* = 1.21; *T* = −7.154, *p* = 0.000, *d* = 1.03; *T* = −6.360, *p* = 0.000, *d* = 1.92; *T* = −5.957, *p* = 0.000, *d* = 1.86; *T* = −8.518, *p* = 0.000, *d* = 1.23 for somatic symptoms, anxiety symptoms, psychosocial functioning, depressive symptoms, and general health, respectively), showing that they were more affected than HWs.

**TABLE 3:** Means and standard deviations on the GHQ-28.

	**Total (*N* = 193)**		**^(a)^HW (*n* = 97)**		**^(b)^HWC (*n* = 96)**		**Comparison**		
				
	***M***	***SD***	***M***	***SD***	***M***	***SD***	***t*_(191)_**	***p***	***Cohen’s d***
Somatic symptoms	2.79	2.37	1.57	1.93	4.03	2.12	−8.445	<.001	1.21*_*L*_*
Anxiety symptoms	3.37	2.47	2.25	2.15	4.51	2.25	−7.154	<.001	1.03*_*L*_*
Psychosocial functioning	1.75	1.8	0.99	1.34	2.51	1.92	*t*_(169,58)_ −6.360	<.001	1.92*_*L*_*
Depressive symptoms	1.43	1.91	0.68	1.43	2.19	2.03	*t*_(170,81)_ −*5.957*	<.001	1.86*_*L*_*
General health TOTAL	9.34	7.40	5.48	5.74	13.23	6.85	−8.518	<.001	1.23*_*L*_*

*^(*a*)^Housewives; ^(*b*)^Housewives caregivers. *N*, null effect size; *S*, small effect size (*d* = 0.20); *M*, medium effect size (*d* = 0.50); L, large effect size (*d* = 0.80).*

After analyzing participant general health and presence of symptomatology, we proceeded to the relationship between these variables and burnout in both groups. As observed in Table 4, the correlation analysis showed that in both HW and HWC, EE was statistically significantly correlated positively to general health (*p* = 0.000), and also to somatic symptoms (*p* = 0.000), anxiety symptoms and insomnia (*p* = 0.000), psychosocial functioning (HW: *p* = 0.000; HWC: *p* = 0.022), and depressive symptoms (*p* = 0.000). In other words, emotional exhaustion is associated with affectation of health. Similarly, DP in both groups was statistically significantly correlated positively with general health (HW: *p* = 0.001; HWC: *p* = 0.001), depressive symptoms (*p* = 0.000), and psychosocial functioning (*p* = 0.000). Moreover, unlike the women in the HW group, in HWC, somatic and anxiety symptoms were associated with depersonalization (*p* = 0.000).

**TABLE 4:** Correlation analysis between burnout syndrome dimensions and general health subscales in the HW and HWC groups.

					**^(a)^HW**										^(b)^HWC		

			**General health**				***Burnout***					**General health**				***Burnout***	

		**S.S.**	**A.I**	**P.F.**	**D.S.**	**G.H.**	**E.E.**	**D.P.**			**S.S.**	**A.I.**	**P.F.**	**D.S.**	**G.H.**	**E.E.**	**D.P.**
^(c)^General	*A*.*I*.	0.67**							General	*A*.*I*.	0.76**						
health		0.000							health		0.000						
	*P*.*F*.	0.68**	0.67**							*P*.*F*.	0.55**	0.58**					
		0.000	0.000								0.000	0.000					
	*D*.*S*.	0.46**	0.49**	0.59**						*D*.*S*.	0.50**	0.57**	0.56**				
		0.000	0.000	0.000							0.000	0.000	0.000				
	*G*.*H*.	0.86**	0.88**	0.86**	0.72**					*G*.*H*.	0.84**	0.88**	0.80**	0.79**			
		0.000	0.000	0.000	0.000						0.000	0.000	0.000	0.000			
^(d)^*Burnout*	*E*.*E*.	0.52**	0.56**	0.45**	0.37**	0.58**			*Burnout*	*E*.*E*.	0.45**	0.46**	0.23*	0.37**	0.46**		
		0.000	0.000	0.000	0.000	0.000					0.000	0.000	0.022	0.000	0.000		
	*D*.*P*.	0.16	0.19	0.28**	0.26**	0.25*	0.31**			*D*.*P*	0.34**	0.35**	0.42**	0.32**	0.43**	0.36**	
		0.125	0.066	0.005	0.009	0.012	0.002				0.001	0.000	0.000	0.001	0.000	0.000	
	*P*.*A*.	−0.02*	–0.18	–0.14	–0.09	–0.20	–0.04	-0.06		*P*.*A*.	−0.24*	–0.19	–0.12	−0.23*	−0.24*	–0.12	−0.21*
		0.032	0.070	0.167	0.416	0.054	0.678	0.543			0.017	0.067	0.251	0.025	0.017	0.037	0.037

**p ≤ 0.01, *p ≤ 0.05. ^(*a*)^Housewives; ^(*b*)^Housewives caregivers; ^(*c*)^General health subscales (S.S., somatic symptoms; A.I, anxiety and insomnia; P.F., psychosocial functioning; D.S., depressive symptoms; G.H., total score in General Health); ^(*d*)^Burnout Syndrome dimensions (E.E., emotional exhaustion; D.P., depersonalization; P.A., personal accomplishment).

PA in the HW group only had a significant negative correlation with somatic symptoms (*p* = −0.032), while in the HWC group, it had a significant negative correlation with somatic symptoms (*p* = −0.017), with depressive symptoms (*p* = 0.025), and with general health (*p* = 0.017). The values of the correlations, which are the effect sizes, are presented in Table 4.

Finally, linear regression analyses were carried out to test the extent to which the different dimensions of the syndrome and the type of activity performed affected general health, and specifically, the presence of emotional disorders (anxiety and depression). The first analysis of the total general health score showed that EE, activity performed, DP, and PA were predictors, in that order. These variables explained 51% (*R*^2^ = 0.51; *f*^2^ = 1.04, large effect size) of affectation of health (*F* = 48.91; *p* = 0.000), and therefore, the regression-line coefficients show that high emotional exhaustion and depersonalization, low personal accomplishment, and caring for a relative with Alzheimer’s explained 51% of affectation of health (see Table 5).

**TABLE 5:** Regression analysis of the influence of burnout variables and activity performed on general health, anxiety symptoms and depressive symptoms.

	**Beta**	**Standard error**	***t***	***p***
**General health**				
Constant	3.638	1.783	2.041	0.043
Emotional exhaustion	0.199	0.030	6.75	0.000**
Depersonalization	0.187	0.063	2.98	0.003**
Personal accomplishment	–0.109	0.043	–2.55	0.011*
Activity performed	5.106	0.835	6.12	0.000**
**Anxiety symptoms**				
Constant	1.772	0.624	2.839	0.005
Emotional exhaustion	0.079	0.010	7.834	0.000**
Personal accomplishment	–0.035	0.015	–2.305	0.022*
Activity performed	1.250	0.298	4.199	0.000**
**Depressive symptoms**				
Constant	–0.556	0.255	–2.178	0.031
Emotional exhaustion	0.039	0.009	4.344	0.000**
Depersonalization	0.054	0.019	2.820	0.005**
Activity performed	1.017	0.255	3.986	0.000**

**p ≤ 0.01, *p ≤ 0.05.

Two regression analyses were done with the same predictor variables to determine the weight of the burnout syndrome and activity performed exerted on the appearance or presence of anxiety and depressive symptoms. The results showed that these variables explained 41.6% (*R*^2^ = 0.416; *f*^2^ = 0.71, large effect size) of appearance or presence of anxiety symptoms (*F* = 46.63; *p* = 0.000). The regression-line coefficients show that high emotional exhaustion, low personal accomplishment, and caring for a family member with Alzheimer’s explained 41.6% of presence of anxiety symptoms (see Table 5).

The variables referred to explained 29.3% (*R*^2^ = 0.293; *f*^2^ = 0.41, large effect size) of the appearance or presence of depressive symptomatology (*F* = 27.56; *p* = 0.000). Activity performed had the highest weight, followed by depersonalization, and finally, emotional exhaustion. Therefore, the regression line coefficients show that high emotional exhaustion, depersonalization, and caring for a family member with Alzheimer’s explained 29.3% of the presence of depressive symptoms (see Table 5).

In view of these results, it could be said that emotional exhaustion, depersonalization, and (lack of) personal accomplishment, along with caring for a family member who is an Alzheimer’s patient, influence health (general state of health, as well as emotional disorders, anxiety, and depressive symptomatology). The weight of these variables differs, both in health (51%) (EE, DP, and low PA) and anxiety symptoms (43.3%) (EE and low PA) and, to a lesser extent, in the presence of depressive symptoms (29.3%) (EE, DP), but emotional exhaustion is the dimension that exerts the most influence, especially in caregivers.

### Primary Analyses

#### Multigroup Structural Equation Modeling

**FIGURE 1:**
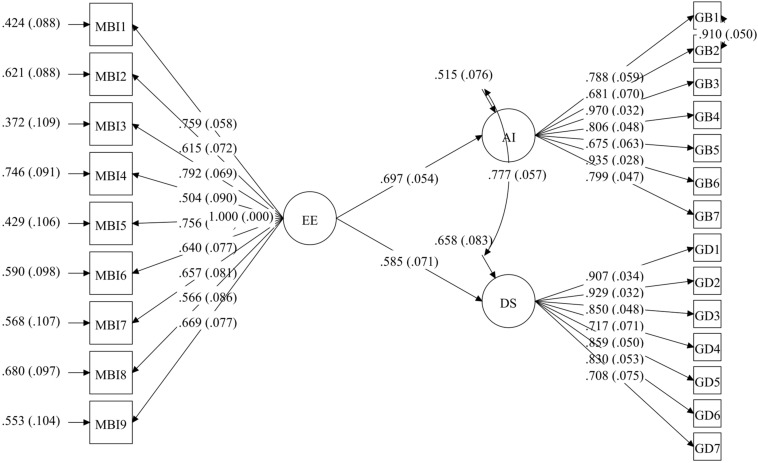
Structural model baseline for the total sample.

We started out with a model made up of three latent variables, emotional exhaustion (with nine quantitative items), anxiety (with seven dichotomous items), and depression (with seven dichotomous items). Our starting hypothesis was that emotional exhaustion influences anxiety and depression and that both dependent variables are correlated. The first estimation of this model yielded a relative fit to the data, RMSEA = 0.06 (*p* = 0.010), CFI = 0.92, TLI = 0.91, WRMR = 1.01. After analyzing the modification index, the correlation between anxiety Items 1 and 2 was 76.56, so it was decided to include this correlation in the general model (see Figure 1). As we can see, the fit indices improved, RMSEA = 0.05 (*p* = 0.340), CFI = 0.95, TLI = 0.94, and WRMR = 0.86. All factor loadings were statistically significant at *p* < 0.001, and standardized loadings ranged from 0.50 to 0.79 for CE, from 0.68 to 0.97 for anxiety and from 0.71 to 0.93 for depression. Then, the model was replicated separately for HW and HWC. In the HW group, this model yielded an adequate fit to the data, RMSEA = 0.04 (*p* = 0.830), CFI = 0.96, TLI = 0.95, and WRMR = 0.81; all factor loadings were statistically significant at *p* < 0.001 and standardized loadings ranged from 0.23 to 0.77 for EE, from 0.57 to 0.93 for anxiety, and from 0.73 to 0.98 for depression. In HWC, the same model yielded an adequate fit to the data, RMSEA = 0.06 (*p* = 0.320), CFI = 0.95, TLI = 0.95, and WRMR = 0.85; all factor loadings were statistically significant at *p* < 0.001 and standardized loadings ranged from 0.47 to 0.85 for EE, from 0.61 to 0.95 for anxiety, and from 0.72 to 0.92 for depression. No modification index was suggested for either group.

**FIGURE 2:**
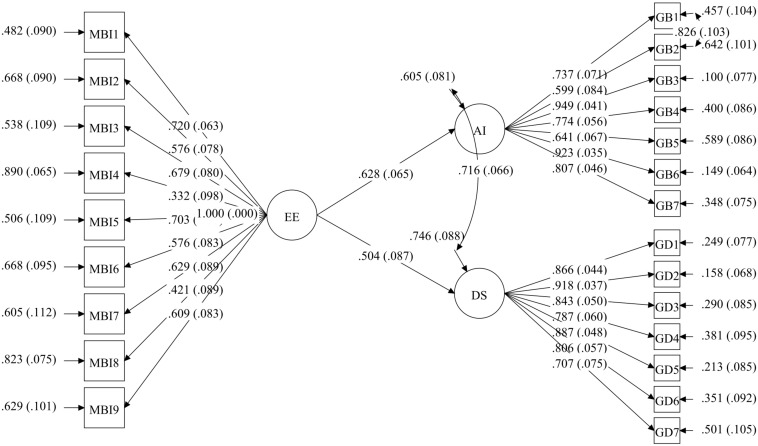
Full invariance multigroup SEM model in housewives (HW).

**FIGURE 3:**
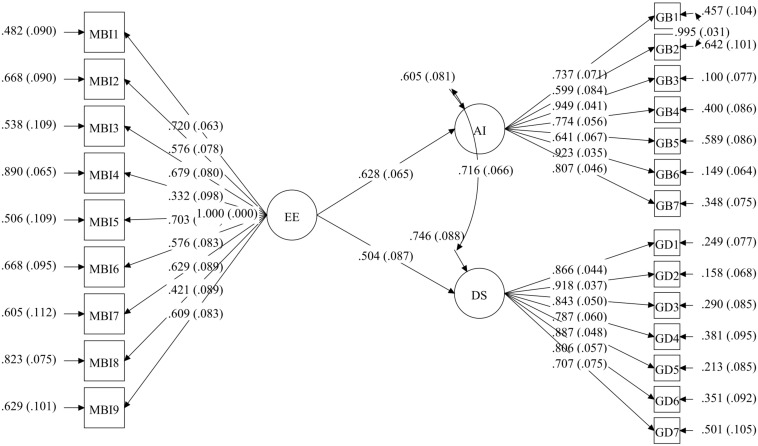
Full invariance multigroup SEM model in housewives who are also family caregivers of Alzheimer’s patients (HWC).

Next, multigroup SEM was examined, starting out from the previous model in which correlation was allowed between Items 1 and 2 of the anxiety scale in both HW and HWC. The fit indices were adequate, RMSEA = 0.05 (*p* = 0.670), CFI = 0.96, TLI = 0.95, and WRMR = 1.2; however, a modification index was found in the HWC group for the correlation between Items 6 and 7 on the depression scale. This correlation was included in the model for this group, setting it at 0 in the HW group, and fit indices were satisfactory: RMSEA = 0.04 (*p* = 0.750), CFI = 0.96, TLI = 0.96, and WRMR = 1.1. No modification index was suggested. As shown in Table 6, the result supported a full invariance hypothesis for groups. When the models were compared with progressive restrictions from the previous model, there were no significant differences in the fit indices. Specifically, the model with factor loading constraints (Model 2) showed an increment in the CFI of 0.010 from the unrestricted model (Model 1); the model that factor variances and covariance constraints were added to (Model 3) showed un increment in the CFI of 0.002 with respect to the model with factor loading constraints (Model 2); the model the regression path constraints were added to (Model 4) showed an increment in the CFI of 0.006 over the model with factor variances and covariance constraints (Model 3); and finally, the model the residual constraints were added to (Model 5) showed an increment in the CFI of 0.001 with respect to the model with regression path constraints (Model 4) (see Table 6). No parameter had to be freed between groups in any of the models to improve fit. The invariant models by group are shown in Figures 2, 3.

**TABLE 6:** Fit statistics for multigroup SEM analysis.

**Model**	**χ^2^**	**DF**	**RMSEA(*p*)**	**CFI**	**TLI**	****Δ** CFI**
Model 1. Unrestricted	534.90**	451	0.04 (0.75)	0.961	0.96	
Model 2. Factor loadings	531.76**	471	0.04 (0.93)	0.971	0.97	0.010
Model 3. Factor variances and covariances	551.97**	495	0.03 (0.95)	0.974	0.97	0.002
Model 4. Regression paths	540.97**	497	0.03 (0.98)	0.980	0.98	0.006
Model 5. Factor residuals	546.68**	506	0.03 (0.99)	0.981	0.981	0.001

**p < 0.01. χ^2^, Chi square value; DF, degrees of freedom; RMSEA, root mean square error of approximation; p, RMSEA significance value; CFI, comparative fit index; TLI, Tucker–Lewis index; Δ, change in statistic. Following Cheung and Rensvold (2002), a ΔCFI < 0.01 indicates that the invariance assumption still holds.

#### Predictors of Emotional Exhaustion

The results of the regression analysis for the HW group showed that hours spent on their tasks and age explained 5.7% (*R*^2^ = 0.057; *f*^2^ = 0.06, small effect size) of EE (*F* = 2.83; *p* = 0.064). Although the hours spent variable was not significant in explaining EE (*p* = 0.719), age was (*p* = 0.020) (see Table 7). When the factorial ANOVA was performed, it was observed that neither of the two variables was significant, nor the number of children living at home (*p* = 0.315; *η^2^_*p*_* = 0.041, small effect size) or the number of chronic diseases (*p* = 0.763; *η^2^_*p*_* = 0.013, small effect size); resulting in a non-significant model (*F* = 0.94; *p* = 0.507), which explained 10.8% (*R*^2^ = 0.108) of EE (see Table 8).

**TABLE 7:** Regression analysis of the influence of hours spent on homework and age on emotional exhaustion.

	***B***	**Standard error**	***t***	***p***
**HW**				
Constant	9.174	5.806	1.580	0.117
Hours spent on homework	0.058	0.162	0.361	0.719
Age	0.246	0.104	2.376	0.020*
**HWC**				
Constant	9.882	7.063	1.399	0.165
Hours spent on homework	0.307	0.171	1.795	0.076
Age	0.381	0.142	2.690	0.008**

*p ≤ 0.05; **p ≤ 0.01.

**TABLE 8:** Factorial ANOVA for the HW and HWC groups.

	**Quadratic mean**	***F***	***p***	***η^2^_*p*_***
**HW**				
Corrected model	134.042	0.939	0.507	
Sons/daughters who live at home	171.050	1.199	0.315	0.041*_*M*_*
Number of chronic diseases	55.171	0.387	0.763	0.013*_*S*_*
**HWC**				
Corrected model	238.203	1.127	0.338	
Sons/daughters who live at home	495.401	2.345	0.081	0.096*_*M*_*
Number of chronic diseases	792.979	3.753	0.015*	0.146*_*L*_*
Relationship	46.796	0.221	0.802	0.007*_*S*_*

*p ≤ 0.05. *N*, null effect size; *S*, small effect size (*d* = 0.01); *M*, medium effect size (*d* = 0.06); L, large effect size (*d* = 0.14).

The results of the regression analysis for HWC showed that hours spent on their tasks and age explained 13.5% (*R*^2^ = 0.135; *f*^2^ = 0.16) of EE (*F* = 7.25; *p* = 0.001). The hours spent variable showed a trend in explaining EE (*p* = 0.076), and age was significant (*p* = 0.008) (see Table 7). The factorial ANOVA showed that the number of children living at home was tendential (*p* = 0.081; *η^2^_*p*_* = 0.096, medium effect size), that the relationship was not significant (*p* = 0.802; *η^2^_*p*_* = 0.007, small effect size), and that the number of chronic diseases was significant (*p* = 0.015; *η^2^_*p*_* = 0.146, large effect size), especially between those women with no chronic diseases and those who had one (*p* = 0.044). In general, the model was not significant (*F* = 1.13; *p* = 0.338), explaining only 31.6% (*R*^2^ = 0.316) of EE (see Table 8).

Therefore, in the HW group, only age predicted EE, and in the HWC group, age and the number of chronic diseases, and hours spent on care and the number of children living at home were tendential.

## Discussion

Our first goal was to determine the prevalence of the burnout syndrome and general health problems and presence of emotional disorders associated with the type of activity performed as HW or HWC.

The prevalence of burnout in this study approached 14.9%, which is similar to other studies (Barragán et al., 2015) done in the healthcare sector in various countries, including Spain. In spite of the few studies devoted to burnout syndrome in housewives, 11.9% prevalence found in this study surpasses the 1.95% found by Pascual (2001), although in our study, the occupational activity of the participants was exclusively in the family setting and was unremunerated. On the contrary, that study included women who not only worked in the family setting but also outside of it, and were remunerated, a factor that could explain the difference in their findings. In our study, housewives were devoted to housework, which in itself can be stressful considering the associated heavy overburden of work, as mentioned, and its lack of social recognition (Moral et al., 2011). These findings are also in line with Rodríguez et al. (2014), who found a burnout prevalence of 11.3% in housewives, which is similar to our sample (11.9%). This is aggravated if in addition, the housewife must care for an Alzheimer’s patient. Management of this situation requires the housewife caregiver to have close interaction with the patient, demanding more dedication (Son et al., 2007; Fernández de Larrinoa et al., 2011) and excessive overburden of work. As the overburden of work increases, so does deterioration of their health (Aldana and Guarino, 2012; Evolahti et al., 2013) more than those who are only working in the home and do not have to take on the role of informal caregivers (Pinquart et al., 2012; Rivera and Requena, 2013). All this could justify in some way the subtle difference in the higher percentage in HWC.

Therefore, the occupation of housewife is associated with the presence of burnout, and this is slightly higher in those who in addition are the main caregiver of a family member with Alzheimer’s disease. The presence of burnout may also lead to severe harm to their health. In fact, in our study, we found that the health of both groups was affected, with the higher percentage among the caregivers. These results are in line with previous research, which has demonstrated affectation of health and repercussion on physical and psychological health of women devoted to informal caregiving in the family setting (Sánchez-Herrero and Sánchez-López, 2011; Suñer-Soler et al., 2013).

Another possible explanation for the higher percentage of affectation of health in HWC may be found in their priority for caring for the family patient, leaving personal projects and self-care on a secondary plane. They often avoid or delay visits to the doctor for their own personal attention as long as possible, even when they feel discomfort or pain. Over time, these ailments may temporarily impede them from working, at which point they are finally compelled to go to healthcare services (Espín, 2009).

In view of all of the above, it might be said that caring for a family member with Alzheimer’s disease is an additional risk factor for the caregiver. We therefore wanted to know to what extent the activity performed and burnout syndrome affected their health (general state of health) and in particular, the presence of emotional disorders (anxiety and depression). The results confirmed the considerable weight exerted by these variables. Emotional exhaustion turned out to be the most influential dimension, especially in caregivers. Our findings with respect to how it affects their general state of health are congruent with previous research on burnout and health (Lovell and Wetherell, 2011; Suñer-Soler et al., 2013). Caregiving work, as mentioned above, is emotionally very demanding, increasing the feeling of overburden, and this effect is strengthened by having to cope with it alone in most families. The lack of social support facilitates the appearance of emotional exhaustion, vulnerability to stress, and deterioration of health (Moral et al., 2011).

Findings concerning mental health, and specifically presence of anxiety and depressive symptoms, are consistent with previous studies. Several authors have confirmed the relationships existing between anxiety and health in women in general, and in particular, in those who only care for the home compared to those who also work outside of it. This finding is also consistent with the results found in our study. Informal caregivers show higher risk of suffering from emotional problems such as anxiety and depression (Tuithof et al., 2015; Méndez et al., 2010), and their health is also more affected.

According to Armon et al. (2014), burnout can be a predictor of anxiety and depressive symptoms in apparently healthy individuals. In caregivers, it would be significantly related to a greater extent with anxiety levels and depersonalization with presence of depressive symptomatology (Yilmaz et al., 2009).

Some studies have shown the role of emotional exhaustion. As mentioned, the presence of depression in caregivers of patients with dementia would in turn be related to the presence of emotional exhaustion (Truzzi et al., 2012). Other studies have found that depressive symptoms are higher in persons with high levels of emotional exhaustion (Chiu et al., 2015). In our study, the effect size in relation to anxiety was higher for the emotional exhaustion and personal accomplishment variables, while in depressive symptomatology, it was emotional exhaustion and depersonalization that showed the larger effect size, demonstrating the importance that emotional exhaustion seems to have along with caregiving in emotional disorders present in housewives. Our findings again show the role of this dimension to be a central axis of the burnout syndrome (Avargues-Navarro et al., 2010; Maslach and Leiter, 2016).

In this study, the importance of EE, anxiety, and depression was confirmed in the sample analyzed, with higher scores in HWC than in HW. It was also confirmed that emotional exhaustion influences anxiety and depression equally and with the same intensity in both groups, although emotional exhaustion is higher in the group of caregivers and there are some differential variables that could explain the amount of exhaustion in the two groups differently. In both groups, being younger would act as a protective factor; however, in HWC, it was also confirmed that the presence of chronic disease could be acting as a risk factor such that caregivers find themselves immersed in a pattern of spiraling exhaustion-illness, making the situation even more severe, and leaving them more vulnerable. In this group, the number of hours spent on care and having children living at home would also affect EE levels tendentially. All of this could explain the higher percentage of women with high levels of exhaustion in the group of caregivers. These findings are in line with what has previously been shown by authors who have identified caregiving in itself as an additional risk factor for developing health problems in the role of housewife (Crespo et al., 2005; Lovell and Wetherell, 2011; Campos-Puente, 2016). That is, in our opinion, and in view of our findings, the work of caring for a family member with Alzheimer is an additional burden that would increase the vulnerability of the housewife to demands intrinsic to her own role as housewife and to its consequences to her health.

In summary, the occupation of housewife has in itself been confirmed as a risk factor for developing stress and burnout, affecting physical and psychological health with anxiety and depression emotional disorders. This is accentuated even more when they must also carry out the role of caregivers of a family member who is an Alzheimer’s patient.

In the light of these results, intervention for preventing burnout and emotional alterations derived from it for HW, and especially, HWC, should include a series of actions (institutional, socio-community, group, and individual) to ensure the efficacy of programs developed. Some of these actions to start work with would be diffusion through the communication media of messages directed at a new image of the housewife, in general, and in particular, of those who are also caregivers, in today’s society, showing the intrinsic values of this study population. Public information campaigns should also be designed for community awareness of the problem these women live with and facilitate their access to the resources necessary to provide them with social and family support. Similarly, direct work with this group, teaching them techniques for caring for patients at different stages of the disease’s development, as well as time management techniques that favor conciliation of caregiving with their participation in social life, favoring the design of their own life projects. Finally, more personalized actions focusing on strengthening personal and psychological resources are necessary to enable these women to manage stress satisfactorily, regulate their emotions, and take care of themselves as well (Campos-Puente, 2016). In this line, future studies should focus on those personal variables that can be modified by intervention and that could act as protectors, and perform a relevant role in adequate coping with the daily stress they live with. This would help design more specific action directed at strengthening personal resources.

Possible limitations of this study are the sampling procedure used. Higher syndrome prevalence figures might have been found if other women who are housewives and housewife caregivers of a relative who is an Alzheimer’s patient who do not attend the association centers had been included, and therefore, who had less time and support. It would have also been of interest to include some measure of caregiver overburden and of perceived support. Another limitation is the small sample size, which implies the need to replicate these analyses in another similar sample to confirm the validity of the conclusions.

## Conclusion

Our findings provide evidence for the need to design burnout prevention intervention programs for housewives who are caregivers for a family member who is an Alzheimer’s patient because the effect of the caregiving task itself and presence of burnout, and more specifically, emotional exhaustion, can have on their physical and emotional health. Although it is true that there are laws protecting caregivers of dependents, they are very limited and do not fit to the specific needs of this group. Today the woman who undertakes the role of informal caregiver in most families continues to set aside her own professional and personal development. When this role disappears, many of these women’s choices are reduced to the single option of caring for the home, either because her educational opportunities or her own physical and psychological health have been lost along the way. There are also many women who are in this situation because of having to care for their children and the home full-time for many years. This study demonstrates the importance of providing support and attention for housewives exposed in their daily routines to conditions favoring stress and burnout, as well as their impact on their health and psychological wellbeing. It would therefore be of interest in future research to undertake the study of variables which can orient specific strategies and personal resources that need to be promoted in these women; in other words, those variables that could influence how they cope with their caregiving tasks, and what would help determine risk factors and protection from developing burnout, in general and in each of its dimensions, and from affecting their physical and emotional health in their personal situation. In view of all of the above, previous studies on burnout, and in particular, those focusing on caregiving, have found that some of these variables could be, for example, the quality of the relationship between the caregiver and the patient before the need for care arose, self-care of the caregiver herself, their feelings of competence, resilience, post-traumatic growth, and the type of coping strategy employed (see Avargues-Navarro et al., 2010; Fernández-Lansac and Crespo, 2011; Hodgins et al., 2011; Díaz and Ponsada, 2017; Navarro-Abal et al., 2017; Pérez-San-Gregorio et al., 2017; Campos-Puente et al., 2019).

## Data Availability Statement

The datasets generated for this study are available on request to the corresponding author.

## Ethics Statement

The studies involving human participants were reviewed and approved by the University of Seville. The participants provided their written informed consent to participate in this study in accordance with the Declaration of Helsinki.

## Author Contributions

MA-N, MB-M, and AC-P: conception and design of the work, bibliography research about the topic, data collection, data analysis and interpretation, drafting the article, revising the article critically for important intellectual content, and giving final approval of the version to be submitted. MS-M: conception and design of the work, data analysis and interpretation, drafting the article, revising the article critically for important intellectual content, and giving final approval of the version to be submitted. MP-S-G and AM-R: conception and design of the work, data analysis and interpretation, revising the article critically for important intellectual content, and giving final approval of the version to be submitted.

## Conflict of Interest

The authors declare that the research was conducted in the absence of any commercial or financial relationships that could be construed as a potential conflict of interest.
